# Radiation‐Induced Cutaneous Tissue Reactions in Neurointerventional Procedures: A Systematic Review of Reported Effects and Associated Dose Levels

**DOI:** 10.1111/1754-9485.70156

**Published:** 2026-07-30

**Authors:** Mohamed Khaldoun Badawy, Fatimah Balasim, Caitlan De Hoedt, Mercer Kim, Samuel Montgomery Lilli, Alice Gutowski

**Affiliations:** ^1^ Monash Radiology Monash Health Clayton Victoria Australia; ^2^ Department of Medical Imaging and Radiation Sciences Monash University Melbourne Victoria Australia; ^3^ Discipline of Medical Radiation, School of Health and Biomedical Sciences RMIT University Melbourne Victoria Australia; ^4^ Library Services Monash Health Melbourne Victoria Australia

**Keywords:** alopecia, embolisation, fluoroscopy, radiation injuries, radiodermatitis

## Abstract

Neurointerventional procedures can deliver high localised skin doses and may cause radiation‐induced cutaneous tissue reactions, yet the doses at which these occur have not been synthesised specifically for this setting. We systematically reviewed reported cutaneous reactions and associated radiation doses after fluoroscopy‐ or digital subtraction angiography‐guided cranial or spinal neurointerventional procedures. Following PRISMA 2020, Ovid MEDLINE, Embase, Scopus and the Cochrane Library were searched in April 2026; case reports, case series and observational studies reporting a cutaneous reaction and/or a relevant skin dose were eligible. Screening and extraction were performed by two independent reviewers, and studies were appraised with the Joanna Briggs Institute checklists; findings were synthesised narratively, with cohort‐derived incidence reported separately from case‐level data. Twenty‐five studies (approximately 686 patients; 11 countries) were included. Alopecia/epilation was the dominant reaction (21 studies); dermatitis/erythema (4) and ulceration/necrosis (2) were less frequent. Among cohorts, incidence ranged from approximately 5% to 56% and rose with skin dose and procedural complexity. Reported onset doses tracked established deterministic thresholds: temporary epilation clustered around the approximately 3 Gy threshold, whereas erythema and ulceration occurred at higher, usually cumulative, doses, and epilation below approximately 2 Gy was reported in radiosensitive patients. Alopecia appeared 2–5 weeks after the procedure and ulceration months to years later. Dose metrics were inconsistent, and 4 of 25 studies reported no dose. Standardised reporting of peak skin dose and dose‐triggered, structured follow‐up is needed.

## 1 Introduction

Neurointerventional radiology (INR) has become central to the management of cerebrovascular disease, offering minimally invasive endovascular treatment of conditions such as intracranial aneurysms and arteriovenous malformations (AVMs) [1]. These techniques, which use catheters navigated under fluoroscopic and digital subtraction angiography (DSA) guidance, allow targeted occlusion or remodelling of cerebral and spinal vasculature while avoiding open surgery, and their use has expanded substantially over the past two decades [1].

Many neurointerventional procedures are lengthy and technically complex, requiring prolonged fluoroscopy, repeated high‐dose DSA acquisitions and, in some cases, multiple treatment sessions. Because the cranial and spinal regions of interest lie within or immediately adjacent to the primary X‐ray beam, and because the occipital scalp in particular may remain in the beam entrance field throughout an intervention, the cumulative peak skin dose delivered to a localised area of scalp can be high [2]. This anatomical and procedural context distinguishes INR from many other fluoroscopically‐guided interventions and creates the potential for clinically significant effects on skin and hair.

When the dose to skin exceeds deterministic (tissue‐reaction) thresholds, effects follow a dose‐ and time‐dependent course. Transient erythema may appear within hours to days, followed by temporary epilation, and, at higher doses, dermatitis, moist desquamation, prolonged erythema, permanent epilation and ultimately dermal atrophy, necrosis or ulceration [2, 3]. Scalp hair follicles are particularly radiosensitive, and temporary or permanent alopecia is among the most commonly reported reactions following cranial neurointervention [4]. The severity, latency and reversibility of these reactions are governed by the magnitude of the localised skin dose, making accurate dose characterisation central to both prediction and management [3].

Established estimates of the dose thresholds for these reactions exist, particularly in International Commission on Radiological Protection (ICRP) Publication 118 and in the review by Balter and colleagues [2, 5]. However, these threshold values are derived largely from radiotherapy and accidental or occupational exposure data, with single‐procedure estimates extrapolated from fractionated exposures by radiobiological conversion. They are not specific to neurointervention, and their direct applicability to the distinctive beam geometry, projections, and dose distributions of INR is uncertain. INR‐specific evidence is instead dispersed across individual case reports and small single‐centre cohorts, with no consolidated synthesis [4, 6].

This fragmentation is compounded by inconsistent dose reporting. Studies variously report peak skin dose or entrance skin dose, reference‐point air kerma or cumulative air kerma, and dose–area product, metrics that are not directly interchangeable [2, 6]. Outcome ascertainment is similarly variable, with differences in the reaction definitions used, the grading instruments applied, and the duration and completeness of follow‐up. Together, these inconsistencies have impeded any consolidated, INR‐specific understanding of which reactions occur, at what doses and over what time course.

To date, no systematic review has synthesised radiation‐induced cutaneous tissue reactions together with their associated dose levels specifically in the neurointerventional setting. Existing reviews of fluoroscopic skin injury are broad and procedure‐agnostic [2, 3], and INR dose surveys have typically characterised dose distributions without relating them to cutaneous outcomes [6]. Individual INR studies have made this link and have questioned the applicability of established thresholds to the scalp: Carrick et al., for example, related calculated peak skin dose to temporary epilation in a followed‐up cohort and concluded that the ICRP approximate threshold for temporary epilation may be too high [4]. Such evidence nonetheless remains confined to single centres and has not been consolidated, which represents a clear evidence gap.

The aim of this systematic review was therefore to synthesise the published evidence on radiation‐induced cutaneous tissue reactions, and the radiation doses associated with them, in adult patients undergoing fluoroscopy‐ or DSA‐guided cranial or spinal neurointerventional procedures. Specifically, the review addressed four questions: (1) What radiation‐induced cutaneous tissue reactions have been reported in patients undergoing neurointerventional procedures? (2) What dose levels are associated with these reactions? (3) What is the time to onset of these reactions? (4) Which procedures are most commonly associated with reactions?

## 2 Methods

### 2.1 Protocol and Registration

This systematic review was conducted and reported in accordance with the Preferred Reporting Items for Systematic Reviews and Meta‐Analyses (PRISMA) 2020 statement [7]. The review was not prospectively registered; an a priori protocol defining the research questions, eligibility criteria, and search strategy was established before screening and is available in Appendix S1.

### 2.2 Eligibility Criteria

Studies were eligible for inclusion if they reported on adult patients undergoing fluoroscopy‐ or DSA‐guided cranial or spinal neurointerventional (endovascular) procedures, such as aneurysm coiling, AVM and dural or spinal arteriovenous fistula embolisation, and intracranial angioplasty or stenting. Eligible studies reported radiation‐induced cutaneous tissue reactions (including alopecia, epilation, erythema, desquamation, dermatitis, ulceration or necrosis) and/or a relevant radiation dose, characterised where reported by peak or entrance skin dose, reference‐point or cumulative air kerma, or dose–area product; a skin‐specific dose was not required for eligibility. Eligible designs comprised case reports, case series and observational studies (prospective or retrospective). Only peer‐reviewed, English‐language publications were included. Studies were excluded if they did not involve neurointerventional procedures; reported only animal or phantom data; concerned paediatric patients; described procedures that were not fluoroscopy‐ or DSA‐guided; addressed radiation therapy rather than interventional fluoroscopy; or reported spinal‐pain or orthopaedic procedures (vertebroplasty, kyphoplasty, facet or epidural injections and nerve‐root blocks). Studies reporting only brain or lens dose, or reporting dose with no associated cutaneous outcome, were also excluded, as were narrative or literature reviews, commentaries, editorials and conference abstracts. Non‐peer‐reviewed sources were excluded.

### 2.3 Information Sources and Search

Four databases were searched: Ovid MEDLINE(R) ALL (1946 to 14 April 2026), Embase (1974 to 13 April 2026), Scopus and the Cochrane Library. All searches were conducted in April 2026. The search strategy combined two concept blocks with the Boolean operator AND: (i) cutaneous radiation effects (alopecia, epilation, radiodermatitis, erythema, hair loss or thinning, anagen effluvium, radiation burn, and radiation skin injury or reaction) and (ii) neurointervention (fluoroscopy, embolisation of fistula, AVM, aneurysm or cerebral, intracranial or cerebrovascular lesions, DSA, endovascular technique, interventional neuroradiology and cerebral angiography). Searches were limited to English‐language and human studies. The full search strategy for each database is provided in Appendix S2. The search strategy was developed in consultation with a medical librarian.

### 2.4 Study Selection

**FIGURE 1:**
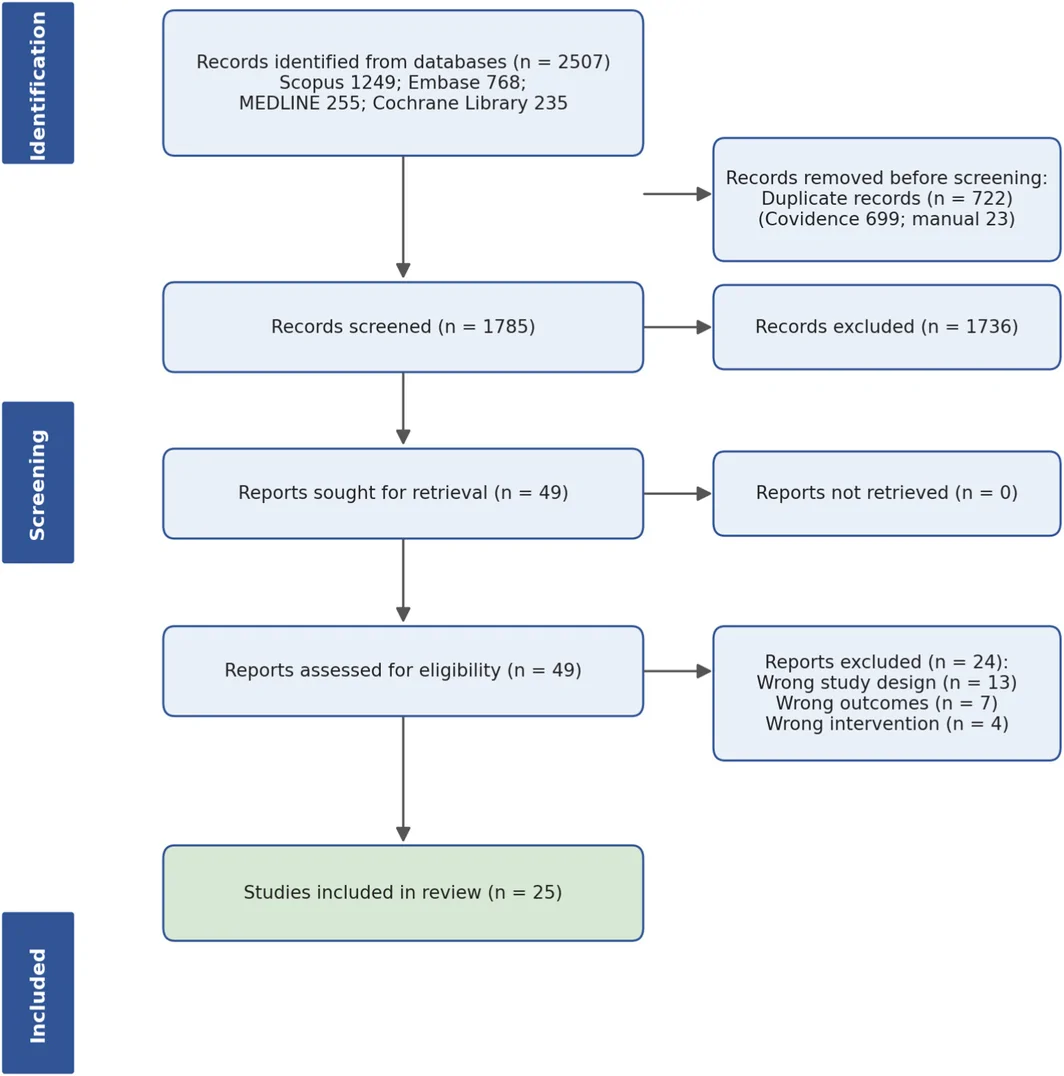
PRISMA 2020 flow diagram of study identification, screening and inclusion. Of 2507 records identified (Scopus 1249, Embase 768, MEDLINE 255, Cochrane Library 235), 722 duplicates were removed; 1785 records were screened, 49 full texts were assessed, and 25 studies were included.

All retrieved records were imported into Covidence systematic review software and deduplicated. Two reviewers independently screened the titles and abstracts of all unique records against the eligibility criteria, and subsequently assessed the full texts of potentially eligible studies. Disagreements at either stage were resolved by discussion or, where consensus could not be reached, by a third reviewer. In total, 2507 records were identified across the four databases (Scopus, *n* = 1249; Embase, *n* = 768; MEDLINE, *n* = 255; Cochrane Library, *n* = 235); 722 duplicate records were removed (699 identified by Covidence and 23 manually), leaving 1785 records for title and abstract screening. Of these, 1736 were excluded and 49 full‐text articles were sought and assessed for eligibility. Twenty‐four full texts were excluded with reasons (wrong study design, *n* = 13; wrong outcomes, *n* = 7; wrong intervention, *n* = 4), leaving 25 studies for inclusion (Figure 1).

### 2.5 Data Extraction

Data were extracted from the included studies using a piloted, standardised data‐extraction form. Extracted fields comprised study identifiers, design and country; population characteristics (sample size, age, sex and clinical indication); intervention details (procedure type, equipment, number of sessions, fluoroscopy and total procedure time, and frame rate); radiation dose (whether dose was reported, the method of determination, the metric used, and the reported value); outcomes (type of cutaneous reaction, assessment tool and time to onset); management; and follow‐up. Two reviewers independently extracted the data and reached agreement; the extracted dataset was subsequently checked against the source publications by the supervising team as a third verification, with any discrepancies corrected against the primary source.

### 2.6 Risk of Bias and Quality Appraisal

The methodological quality of included studies was appraised using the Joanna Briggs Institute (JBI) critical appraisal checklists appropriate to each study design, namely the JBI checklists for case reports and for case series, and the JBI checklist for cohort and analytical observational studies [8]. Two reviewers conducted the appraisal independently, and disagreements were resolved by consensus.

### 2.7 Data Synthesis

A meta‐analysis was not considered appropriate given the substantial clinical and methodological heterogeneity across studies and the predominance of case reports. Findings were therefore synthesised narratively, informed by the Synthesis Without Meta‐analysis (SWiM) guidance, and presented in tabular form [9]. Incidence and denominator‐based estimates were derived only from cohort studies and reported separately from case‐level descriptive data drawn from case reports and selected case series; the latter characterise reaction type, dose at onset, latency and management but cannot yield incidence estimates. Because peak skin dose and entrance skin dose, reference‐point air kerma and cumulative air kerma, and dose–area product are not directly interchangeable, doses were reported by metric without conversion. Reported onset doses were compared descriptively against established deterministic thresholds (ICRP Publication 118; Balter et al.) [2, 5]. Where a study's analytic role (case report, selected case series or denominator cohort) was not clear‐cut, the classification was resolved by consensus between the reviewers.

## 3 Results

### 3.1 Study Characteristics

Twenty‐five studies were included, reporting on approximately 686 patients across 11 countries (most frequently the USA, *n* = 5). Radiation dose was reported in 21 of 25 studies (84%). For synthesis, studies were grouped by their analytic role into case reports (*n* = 13), small case series (*n* = 4: Cho, Mooney, Lv and Nannapaneni) and denominator cohorts (*n* = 8: Carrick, Choi, Corrigall, Ishiguro, Livingstone, Peterson, Perry and Suzuki). Detailed characteristics are presented in Table 1; methodological quality is summarised in Table S1 (JBI critical appraisal).

**TABLE 1:** Characteristics of included studies (*n* = 25).

Study	Country	Design	N	Procedure	Reaction	Radiation dose (metric: value)	Time to onset	Follow‐up
Allen 2009 [10]	USA	Case report	1	Spinal AVM/AVF embolisation	Ulceration/necrosis	Cumulative ~9.3–18.8 Gy (estimated)	17 months (wound)	Yes
Carrick 2024 [4]	Australia	Retrospective cohort	45*	Mixed INR	Alopecia/epilation	Mean PSD 4.2 Gy (3.0–7.0) with epilation vs. 4.6 Gy (3.0–11.1) no effect	~3 weeks	Yes
Carstens 1996 [11]	USA	Case report	1	Spinal AVM/AVF embolisation	Dermatitis/erythema	ESD 24.7 Gy cumulative	4 weeks	Yes
Chen 2025 [12]	China	Case report	1	Cerebral AVF embolisation	Alopecia/epilation	Not reported	3 weeks	Yes
Choi 2019 [13]	South Korea	Retrospective cohort	151	Aneurysm coiling	Alopecia/epilation	CD mean 9.3 Gy; cut‐off 4.44 Gy	mean 18.5 days	Yes
Cho 2014 [14]	South Korea	Prospective series	10	Mixed	Alopecia/epilation	Not reported	mean 3.4 weeks	Yes
Corrigall 2020 [15]	UK	Retrospective cohort	124	Mixed INR	Alopecia/epilation	ESAK; ~50% thinning > 4.5 Gy	2–8 weeks	Yes
Hayakawa 2010 [16]	Japan	Case report	1	Dural AVF (transvenous) embolisation	Alopecia/epilation	ESD 4.2 Gy (measured)	~2 weeks	Yes
Huda 1994 [17]	USA	Case report	1	Cerebral AVM embolisation	Alopecia/epilation	ESD 6.6 Gy (phantom est.)	~5 weeks	Yes
Ishiguro 2021 [18]	Japan	Retrospective cohort	92	Dural AVF embolisation	Alopecia + dermatitis	Ka,r median 6.8 Gy	not stated	Yes
Livingstone 2003 [19]	India	Retrospective cohort	39	Mixed cerebral	Alopecia/epilation	ESD 7.99 & 10.44 Gy (alopecia cases)	4 & 6 months	Partial
López Aventín 2012 [20]	Spain	Case report	1	Aneurysm coiling	Ulceration/necrosis	Not reported (not retrievable)	Alopecia early; chronic ulcer years later (~2 years before presentation)	Yes
López V 2012 [21]	Spain	Case report	1	Cerebral AVM embolisation	Alopecia/epilation	> 3 Gy (estimated)	~3 weeks	Yes
Lv 2010 [22]	China	Retrospective series	7	Dural AVF embolisation	Alopecia/epilation	Not reported	not stated	Yes
Marinello 2016 [23]	Italy	Case report	1	Spinal AVF embolisation	Dermatitis/erythema	3.7 Gy dorsal (9.6 Gy arm)	4 weeks	Yes
Martí 2008 [24]	Spain	Case report	1	Aneurysm embolisation	Alopecia/epilation	> 3 Gy (estimated)	2 weeks	Yes
Mooney 2000 [25]	UK	Retrospective series	3	Cerebral AVM embolisation	Alopecia + erythema	ESD 4–7 Gy single; 9–25 Gy cumulative	overnight–2 months	Partial
Nannapaneni 2007 [26]	UK	Case series	2	Aneurysm coiling	Alopecia/epilation	ESD 3.83 & 4.07 Gy	~1 month	Yes
Perry 2019 [27]	USA	Retrospective cohort	49*	Mixed	Alopecia/epilation	ESD 5.5–19.7 Gy (hair‐loss pts)	not stated	Yes
Peterson 2013 [28]	USA	Retrospective cohort	48	Mixed	Alopecia/epilation	ESD median 3.01 Gy (interventional)	days–weeks	Yes
Suzuki 2008 [29]	Japan	Prospective cohort	103	Mixed neuroembolisation	Alopecia/epilation	ESD mean max 1.9 Gy (measured)	2 weeks–3 months	Yes
Thorat 2007 [30]	Singapore	Case report	1	Aneurysm coiling (GDC)	Alopecia/epilation	ESD ~2 Gy (estimated)	~3 weeks	Yes
Vaccaro 2015 [31]	Italy	Case report	1	Cerebral AVM embolisation	Alopecia/epilation	3.92 Gy (estimated)	~4 weeks	Partial
Wen 2003 [32]	Taiwan	Case report	1	Cerebral AVM embolisation (staged)	Alopecia/epilation	> 3 Gy (estimated)	4–24 days	Yes
Zhang 2020 [33]	China	Case report	1	Basilar angioplasty/stenting	Alopecia/epilation	~1.5 Gy cumulative	week 3 → week 11	Yes

*Note:* Dose metrics are not directly comparable across studies.

Abbreviations: AVF, arteriovenous fistula; AVM, arteriovenous malformation; CD, cumulative dose; ESAK, entrance‐surface air kerma; ESD, entrance skin dose; GDC, Guglielmi detachable coil; INR, neurointerventional radiology; Ka,r, reference‐point air kerma; PSD, peak skin dose.

* Carrick: 45‐patient follow‐up cohort (140 screened with PSD > 3 Gy); Perry: 49‐patient cohort screened with Ka,r > 5 Gy. Cho, Mooney, Lv and Nannapaneni are the case series and are not used for incidence; Perry is treated as a dose‐screened cohort. Full extracted data are provided in Table S2. Martí 2008: onset stated as 2 weeks in the text and 2 months in the figure caption; the text value (2 weeks) is used here.

### 3.2 Reaction Types

**FIGURE 2:**
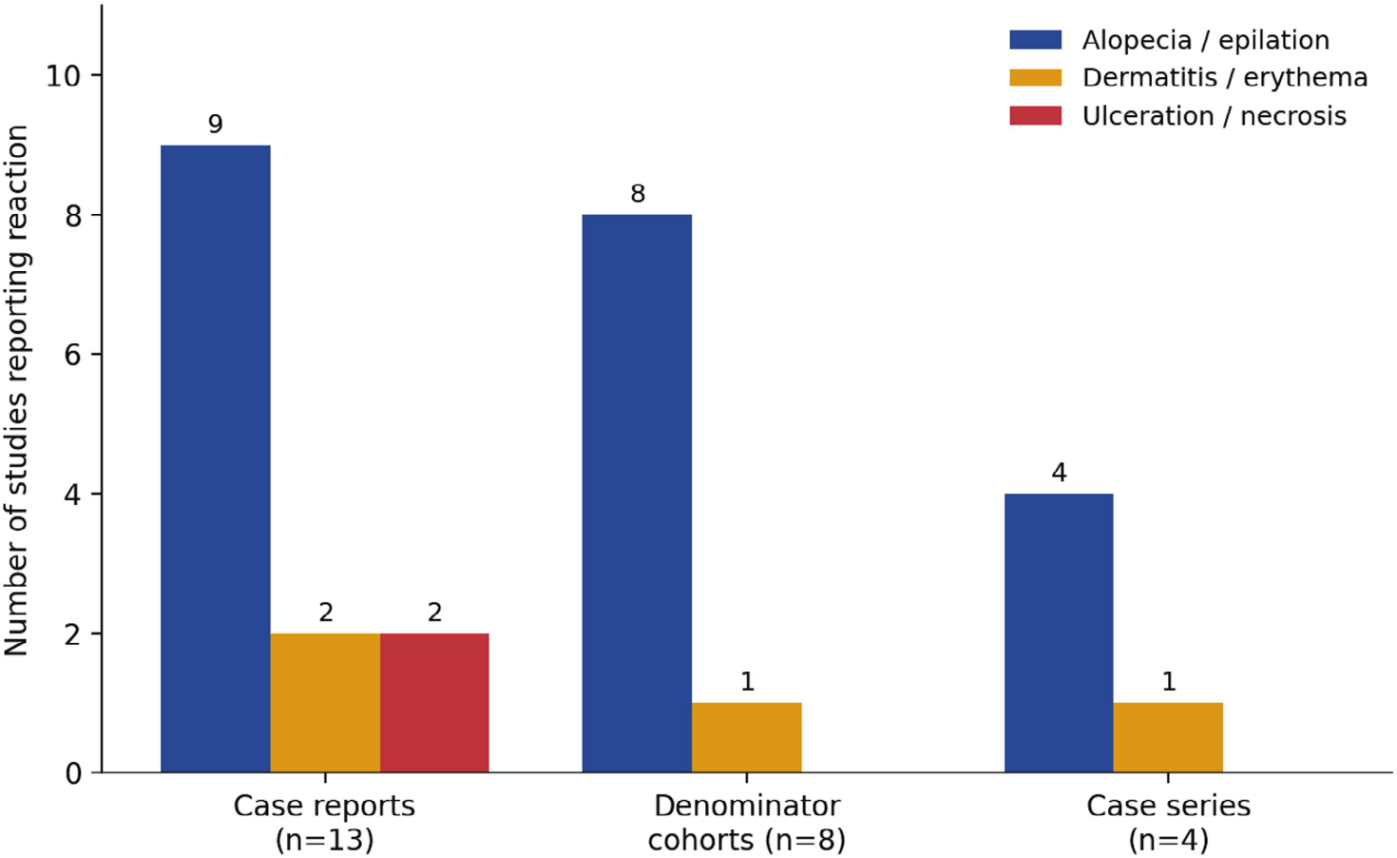
Reported reaction types by study class (case reports, denominator cohorts and case series). Bars show the number of studies reporting each reaction; some studies reported more than one. Frequencies are descriptive and, given the predominance of case reports, should not be read as representative proportions.

Alopecia/epilation was the dominant reaction, reported in 21 of 25 studies; dermatitis/erythema was reported in 4 (Carstens, [11] Marinello, [23] Ishiguro, [18] Mooney [25]), and ulceration/necrosis in 2 (Allen, [10] López Aventín [20]) (Figure 2). Because case reports are selected for notable presentations, reaction‐type frequencies are descriptive and should not be read as representative proportions.

### 3.3 Radiation Dose and Incidence of Reactions

**FIGURE 3:**
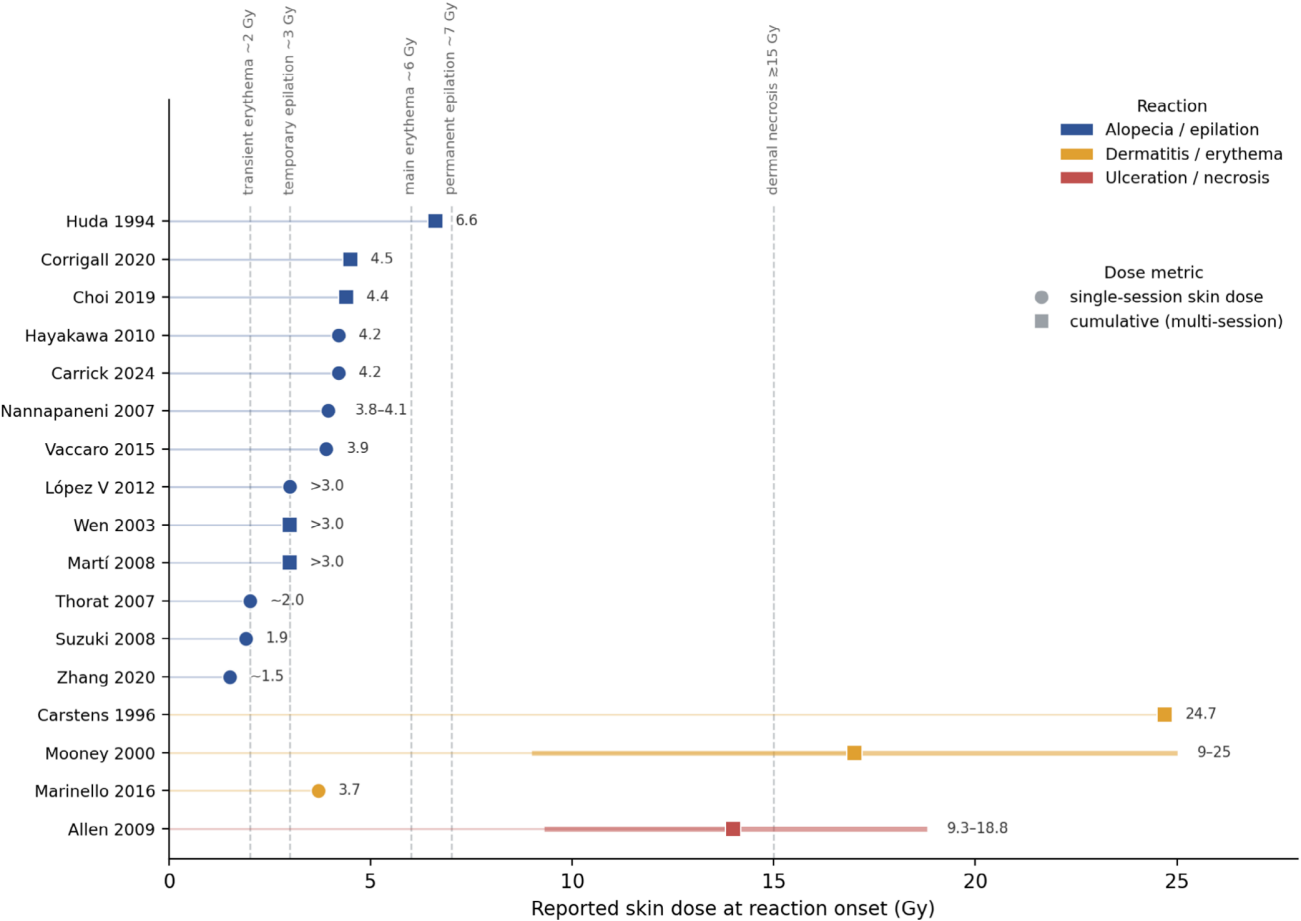
Reported skin dose at reaction onset for each study reporting a dose, grouped by reaction type, against approximate single‐delivery peak‐skin‐dose thresholds (dashed lines; ICRP Publication 118 [5]; Balter et al. [2]). Marker shape denotes the dose metric: Circles, single‐session peak or entrance skin dose (comparable to the thresholds); squares, cumulative dose across multiple sessions (not directly comparable); horizontal bars show reported ranges. Values are shown to one decimal place, with qualifiers retained (~, >). Cohort‐derived points are thresholds rather than individual onset doses (Choi, [13] cumulative‐dose cut‐off; Corrigall, [15] air kerma above which ~50% reported thinning; Suzuki, [29] cohort mean maximum ESD; Carrick, [4] mean peak skin dose in affected patients). Studies without a reported dose (including López Aventín et al. [20]), and cohorts reporting only a median or a range accross patients (Ishiguro [18], Livingstone [19], Peterson [28], Perry [27]), are not plotted. Doses are plotted in the metric reported by each study and are not all peak skin dose; the metric for each study is given in Table 1.

Across the cohorts, the reported incidence of cutaneous reactions ranged from approximately 5% to 56% (Table 2). This wide range largely reflects how each cohort was defined rather than true biological variation alone: incidence was generally lowest in whole‐cohort series that counted all patients irrespective of dose and highest in subgroups pre‐selected for high doses, and it rose with the recorded radiation dose (reported variously as entrance skin dose, peak skin dose or reference‐point air kerma) and with procedural complexity. In whole‐cohort series the proportions were lower (Choi [13] 18/151, 11.9%; Suzuki [29] 6/103, 5.8%; Ishiguro [18] 26/92, 28.3%; Livingstone [19] 2/39, 5.1%), whereas dose‐screened subgroups were higher (Peterson [28] 19/48, 39.6%, entrance skin dose > 2 Gy; Perry [27] 5/49, 10.2%, reference air kerma > 5 Gy; Carrick [4] 25/45, 56%, peak skin dose > 3 Gy), and Corrigall [15] described a graded dose–response, with reported skin or hair effects in 7.7% of patients exposed to 3.0–3.5 Gy, rising to approximately 50% of those exposed above 4.5 Gy. In Peterson's series the median entrance skin dose for the interventional procedures was 3.01 Gy (interquartile range 1.89–4.22 Gy) [28]. Reported onset doses broadly tracked established deterministic thresholds (Table 3; Figure 3): temporary epilation clustered around the ~3 Gy threshold (most case‐report estimates > 3 Gy; cohort signals at Choi [13] cumulative‐dose 4.44 Gy and Corrigall [15] ~4.5 Gy), whereas erythema/dermatitis and ulceration/necrosis occurred at higher, typically cumulative, multi‐Gy doses. Sub‐threshold epilation (~1.5–2 Gy) was reported in a patient with diabetes (Zhang [33]) and one further low‐dose case (Thorat [30]), consistent with individual radiosensitivity.

**TABLE 2:** Incidence of cutaneous reactions in the denominator cohorts (case reports and selected case series excluded).

Study	Procedure	N	With reaction	Incidence	Note
Choi 2019 [13]	Aneurysm coiling	151	18	11.9%	Consecutive; dose‐predictive cut‐offs derived
Suzuki 2008 [29]	Mixed neuroembolisation	103	6	5.8% (7.6% of 79 followed)	Measured ESD; all six aneurysm cases
Ishiguro 2021 [18]	Dural AVF	92	26	28.3%	Erythema and/or alopecia; long operations
Peterson 2013 [28]	Mixed (> 2 Gy subgroup)	48	19	39.6%	Denominator = evaluable patients with ESD > 2 Gy
Livingstone 2003 [19]	Mixed cerebral	39	2	5.1%	Both affected patients in high‐dose group
Corrigall 2020 [15]	Mixed INR	124	dose‐conditioned	~50% above 4.5 Gy	Hair thinning in ~50% exposed > 4.5 Gy
Carrick 2024 [4]	Mixed INR (> 3 Gy followed)	45	25	56% (> 3 Gy subgroup)	Dose‐screened subgroup; epilation 25, erythema 2
Perry 2019 [27]	Mixed INR (Ka,*r* > 5 Gy)	49	5	10.2%	Dose‐screened subgroup; QI cohort

*Note:* Case series (Cho, Mooney, Lv, Nannapaneni) do not provide valid incidence denominators and are excluded. Dose‐screened subgroups (Carrick, Peterson, Perry, Corrigall) report conditional incidence above a dose trigger, not whole‐population incidence.

**TABLE 3:** Reported skin dose at reaction onset versus established single‐delivery deterministic thresholds (ICRP 118; Balter et al.).

Reaction	Threshold (single‐delivery PSD)	Doses reported in this review	Comment
Transient erythema	~2 Gy	Low end of cohort ranges	Consistent
Temporary epilation	~3 Gy	Most case‐report estimates > 3 Gy; cohort signals: Choi 4.44 Gy [13] (cumulative dose), Corrigall ~4.5 Gy [15]; Suzuki mean max 1.9 Gy [29]	Consistent; clusters around/above 3 Gy
Temporary epilation, sub‐threshold	< ~3 Gy	Zhang ~1.5 Gy [33] (diabetic); Thorat ~2 Gy [30]	Individual radiosensitivity
Main erythema/dermatitis	~6 Gy and above	Marinello 3.7 Gy [23] (single); Carstens 24.7 Gy [11] and Mooney 9–25 Gy [25] (cumulative)	Often cumulative, multi‐session
Permanent epilation	~7 Gy	Implied in high‐cumulative‐dose cases	Rarely reported explicitly
Dermal necrosis/chronic ulceration	≈15–18 Gy (late/ischaemic dermal necrosis); ~24 Gy (secondary ulceration)	Allen 9.3–18.8 Gy [10] (cumulative); López Aventín [20] dose not retrievable	High cumulative dose; latency months–years

*Note:* Threshold values are single‐delivery peak skin doses. Values in the ‘Doses reported in this review’ column are given in the metric reported by each study (peak or entrance skin dose, cumulative dose, or air kerma); where a study did not specify the metric, the value is reproduced as reported and should not be assumed to be peak skin dose. Reported doses therefore use heterogeneous, non‐interchangeable metrics and single‐ or multi‐session exposures, so comparison with single‐delivery thresholds is indicative only (see Figure 3).

### 3.4 Time to Onset

**FIGURE 4:**
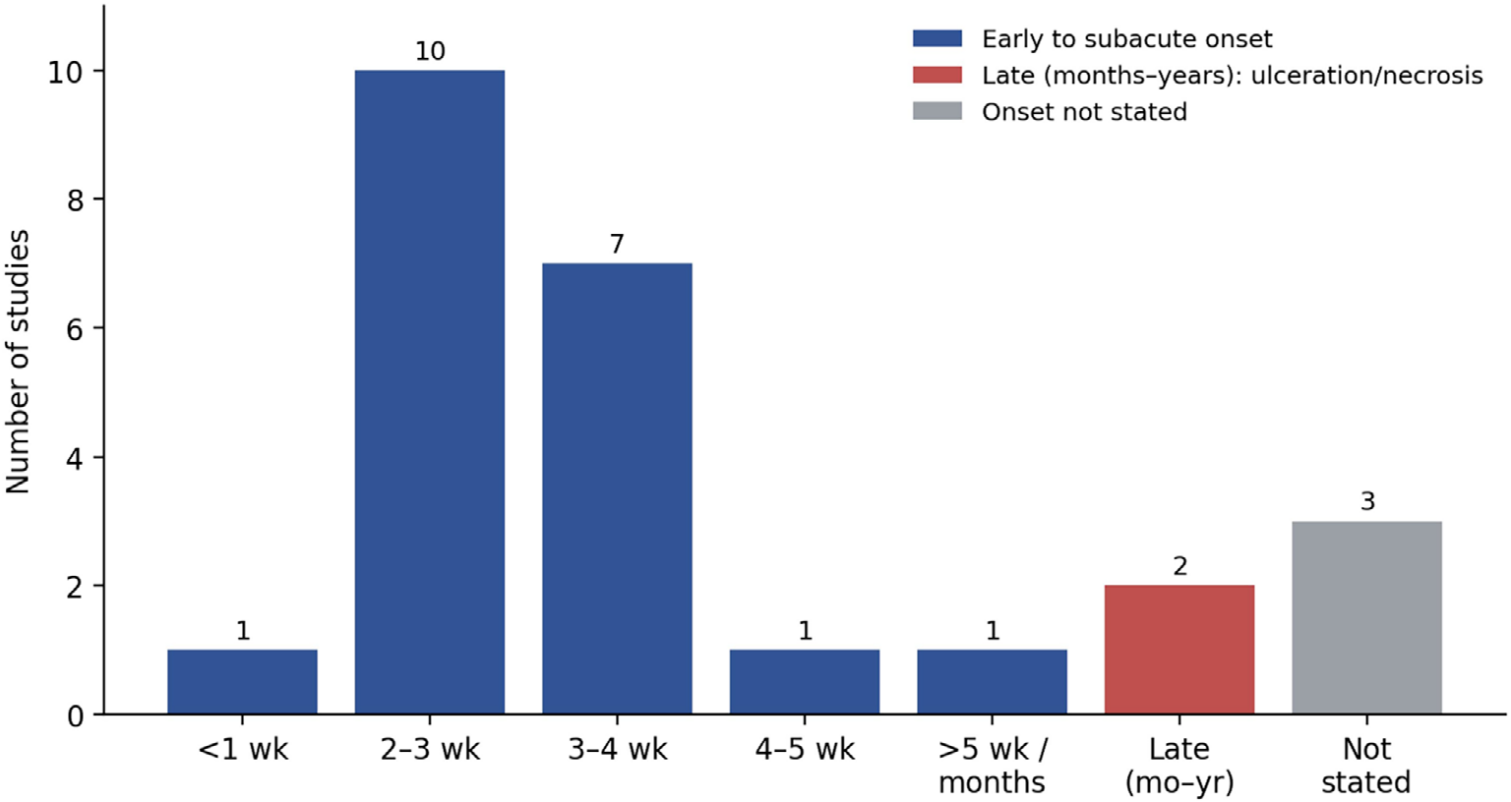
Time to onset of radiation‐induced cutaneous reactions across the included studies. Bars are coloured by onset category: Navy, early to subacute onset; red, late (months to years) ulceration or necrosis; grey, onset not stated. Alopecia and epilation typically appeared 2–5 weeks after the procedure (most commonly ~3 weeks), consistent with radiation anagen effluvium. The ‘late (months–years)’ category comprises the two delayed ulceration/necrosis cases: a radiation burn recognised at 17 months (Allen et al. [10]) and a chronic scalp ulceration that developed within a persistent post‐procedural alopecic patch and presented years later (López Aventín et al. [20]); in the latter the initial alopecia appeared early and only the ulceration was delayed.

Alopecia and epilation typically appeared 2–5 weeks after the procedure (most commonly around 3 weeks; for example, a mean of 18.5 ± 5.3 days in Choi's cohort [13]), consistent with radiation anagen effluvium and with the early (2–8 week) onset window for temporary epilation described in established reference works [2, 5], whereas erythema and dermatitis presented within days to weeks. Ulceration and necrosis were late effects: a radiation burn was recognised 17 months after the procedure (Allen et al. [10]), and a chronic scalp ulceration developed within a persistent post‐procedural radiation‐damaged alopecic patch and presented years later, well beyond the usual follow‐up window (López Aventín et al. [20]; Figure 4).

### 3.5 Procedures Associated With Reactions

Reactions were most frequently reported after AVM/AVF embolisation (cerebral AVM/AVF, *n* = 6 studies; spinal AVM/AVF, *n* = 3; dural AVF, *n* = 3) and aneurysm coiling (*n* = 5), with a further 6 ‘mixed’ INR cohorts and single reports after neuroembolisation and angioplasty/stenting. The pattern reflects long, complex, often multi‐session embolisation procedures, with dural and spinal arteriovenous fistula work and prolonged operative times particularly associated with higher doses and reactions.

## 4 Discussion

Across 25 studies and approximately 686 patients, temporary alopecia (epilation) was by far the most frequently reported radiation‐induced cutaneous reaction after neurointerventional procedures, followed by erythema or radiodermatitis and, least often, ulceration or necrosis. Reactions occurred most commonly after the longest and most complex embolisation procedures (cerebral and spinal arteriovenous malformation and dural arteriovenous fistula embolisation, and aneurysm coiling), and the severity and latency of effects broadly tracked the magnitude and cumulative nature of the skin dose. The evidence base, however, was dominated by single case reports, and dose was reported using a heterogeneous and non‐interchangeable mix of metrics, which together constrain what can be concluded.

Where peak or entrance skin dose was reported, the doses associated with temporary epilation clustered around the approximately 3 Gy threshold conventionally cited for this effect, and below the approximately 7 Gy threshold for permanent epilation (ICRP Publication 118; Balter et al.) [2, 5]. This was reinforced by the larger cohorts: Choi et al. [13] derived a cumulative‐dose cut‐off of 4.44 Gy for temporary alopecia, Corrigall et al. [15] observed hair thinning in approximately half of patients exposed above 4.5 Gy, and Suzuki et al. [29] recorded a mean maximum entrance skin dose of 1.9 Gy with epilation confined to higher‐dose patients. Erythema, radiodermatitis and ulceration were reported at higher and typically cumulative multi‐Gy doses (e.g., 9–25 Gy cumulative in the cases of Mooney et al. [25] and Allen et al. [10]). The neurointerventional literature is therefore broadly consistent with deterministic thresholds derived predominantly from cardiac and general fluoroscopically‐guided interventions, lending those thresholds external validity in this setting, while underlining that the scalp, frequently within the entrance beam, is the critical structure in cranial work.

Several reports described reactions at or below the nominal epilation threshold: temporary alopecia after an estimated ~1.5 Gy in a patient with type 2 diabetes (Zhang et al. [33]) and after ~2 Gy following prolonged single‐projection coiling. Carrick et al. [4] made the same case from cohort data, concluding that the ICRP approximate threshold for temporary epilation may be too high for the scalp; when follow‐up was extended to patients with a calculated peak skin dose of 2–3 Gy over a six‐month trial, 3 of 10 contacted patients reported some degree of epilation. Diabetes (also noted by Allen et al. [10]), repeated or staged procedures with cumulative dosing, short inter‐session intervals, fixed beam projection, and equipment underperformance (an image‐intensifier fault contributing to the doses reported by Mooney et al. [25]) recurred as modifying factors. These observations are consistent with recognised determinants of individual radiosensitivity and argue against treating threshold values as guarantees of safety, particularly in patients with diabetes or connective‐tissue disease.

The most consistent finding of this review was inconsistency. Dose was not reported at all in 4 of 25 studies, and where reported was expressed variously as peak or entrance skin dose, reference‐point or cumulative air kerma, or dose–area product, metrics that are not directly interchangeable without assumptions about beam geometry, backscatter and field size. Even where air kerma is reported, the displayed value itself carries an accuracy tolerance of approximately ±35% under international (IEC) standards, which adds further uncertainty and may contribute to the apparent variation in dose between systems. System configuration is a further source of heterogeneity: studies seldom stated whether a single‐plane or biplane system was used, or whether a reported dose referred to a single plane or to the combined exposure from both planes. Outcome ascertainment ranged from systematic clinical review with dermoscopy and biopsy to retrospective telephone self‐report, the latter likely to underestimate milder effects. This heterogeneity precluded meta‐analysis and the derivation of pooled, metric‐specific thresholds, and is itself a key result: the field cannot yet answer its own central question, the dose at which reactions occur, with the precision that standardised reporting would allow. Routine recording and publication of peak skin dose, alongside air kerma and dose–area product, and the use of a consistent cutaneous‐reaction grading scheme, would materially improve future synthesis. Reports should also state how peak skin dose was obtained, whether machine‐reported, derived from dose‐management or skin‐dose‐mapping software, or independently calculated, because these sources are not equivalent and standardisation of the method is as important as standardisation of the metric. At a minimum, studies should report the standardised machine‐derived dose indices (reference‐point air kerma, dose–area product and fluoroscopy time) and, where feasible, provide the underlying per‐irradiation‐event data as anonymised supporting information, so that peak skin dose can be estimated consistently and compared across studies.

Radiation‐induced alopecia is probably under‐recognised and is readily mislabelled, including as alopecia areata; the characteristic geometric, sharply demarcated patch corresponding to the beam‐entry site, together with the procedural history and dermoscopic features, distinguishes it (Vaccaro et al. [31]; Cho et al. [14]). The method of ascertainment mattered: self‐reported cohorts such as Carrick et al. [4] are likely to undercount effects relative to a structured review. Late injuries were especially easily missed: a radiation burn was recognised only 17 months after the procedure (Allen et al. [10]), and a deep scalp ulceration developed years later within a persistent, post‐procedural radiation‐damaged alopecic patch and presented only once the ulcer itself had been present for around two years (López Aventín et al. [20]). Such late, evolving injuries arise long after the 2–5‐week window in which epilation appears, so follow‐up adequate for epilation will not capture late necrosis or ulceration.

These findings support a dose‐triggered, structured follow‐up pathway after neurointerventional procedures, analogous to the substantial‐radiation‐dose‐level (SRDL) sentinel‐dose programmes recommended for fluoroscopically‐guided interventions [34] (e.g., peak skin dose ≥ 3 Gy or reference‐point air kerma ≥ 5 Gy). Three points warrant emphasis. First, the quantities recorded by most systems are reference‐point air kerma and dose–area product, yet it is the peak skin dose that determines the risk of a tissue reaction; because the irradiated skin in neurointervention is the scalp, which often lies within the entrance field across several projections, these recorded quantities may substantially over‐ or under‐estimate the localised peak skin dose, so peak skin dose should be recorded or estimated for every high‐dose case rather than relying on the air‐kerma readout alone. For such high‐dose cases, peak skin dose estimation is best performed or overseen by an appropriately qualified medical physicist [34], given the several corrections required to convert the recorded air kerma into a localised skin dose, including inverse‐square geometry, table and pad attenuation, air‐kerma‐to‐tissue‐dose conversion and backscatter [35, 36]; studies that report a calculated peak skin dose should also document which of these corrections were applied, so that the values can be interpreted and compared across studies. Second, because reactions, including epilation, were reported at or below approximately 2 Gy in patients with heightened radiosensitivity, a lower trigger, or at least heightened vigilance for at‐risk patients, may be warranted rather than reliance on generic thresholds; recognised risk factors include diabetes mellitus, hyperthyroidism, genetic radiosensitivity syndromes such as ataxia‐telangiectasia, and concurrent radiosensitising drugs [2]. Third, follow‐up must be timed to the natural history of the reactions: clinical review at approximately 2–4 weeks to detect epilation and erythema, a further review at around 10–12 weeks, and review at 6–12 months for patients who received high cumulative or repeated exposures, with a low threshold for dermatological referral. Wherever feasible, assessment and grading of suspected reactions should be undertaken by a dermatologist experienced in radiation‐induced skin effects rather than by the proceduralist alone, to improve diagnostic accuracy and to standardise the grading and reporting of these reactions. This matters because late ulceration and necrosis can present from roughly ten weeks to more than a year after exposure. Patients should be counselled about transient hair loss, and the possibility of other skin reactions, before discharge, and the dose‐reduction strategies repeatedly recommended in the included reports (collimation, pulsed fluoroscopy, reduced frame rates, variation of gantry angulation to avoid a fixed beam‐entry site, additional beam filtration and real‐time skin‐dose monitoring) remain the principal means of prevention.

Strengths of this review include a thorough, librarian‐developed search across four databases, duplicate independent screening, extraction verified against the source publications, formal JBI critical appraisal, and the deliberate separation of cohort‐derived incidence from case‐level description. Several limitations temper the conclusions. The evidence is dominated by case reports, which are selected for notable or severe presentations and cannot yield incidence estimates; reported reaction‐type frequencies are therefore descriptive only. Restriction to English‐language publications may have excluded relevant cases. Heterogeneity of dose metrics prevented quantitative pooling. The JBI appraisal showed that, although the case reports were generally well documented, the cohort studies were commonly limited by an absence of adjustment for confounding and of strategies for incomplete follow‐up, and by reliance on self‐reported outcomes; one small case series (Lv et al. [22]) reported the cutaneous outcome only incidentally and without dosimetry. Reported incidence across the unselected or dose‐screened cohorts spanned roughly 5%–56%, reflecting these methodological differences as much as true variation. Finally, the dose comparison summarised in Figure 3 and Table 3 necessarily juxtaposes non‐equivalent dose quantities (peak or entrance skin dose, cumulative multi‐session dose, and air‐kerma‐derived values) and single versus multi‐session exposures against single‐delivery thresholds; these comparisons are therefore indicative only and must not be read as quantitative equivalence between studies.

The priority is a prospective, multicentre observational cohort that records standardised peak skin dose for every neurointerventional procedure and applies structured, dose‐triggered follow‐up: full cutaneous assessment for patients exceeding a defined skin‐dose trigger, with lighter review of those below it so that the full dose–response relationship, and a true population denominator, are preserved rather than truncated. Coupled with consistent outcome grading, such data could define neurointervention‐specific reaction thresholds, estimate true incidence and support predictive dose models. Automated dose tracking (radiation dose structured reports and skin‐dose mapping software) now makes this achievable at low marginal cost.

## 5 Conclusion

Radiation‐induced cutaneous tissue reactions after neurointerventional procedures are predominantly temporary alopecia, appearing around three weeks after exposure at peak skin doses near the approximately 3 Gy epilation threshold, with erythema, radiodermatitis and, uncommonly, ulceration or necrosis occurring at higher, usually cumulative, doses and occasionally below threshold in radiosensitive patients. The reactions reported in neurointerventional practice are broadly consistent with established deterministic thresholds, but heterogeneous dose reporting and an evidence base dominated by case reports preclude firm, metric‐specific thresholds and reliable incidence estimates. Standardised peak skin dose reporting, consistent outcome grading, and a dose‐triggered, structured follow‐up pathway that extends beyond the epilation window to detect late injury are the priorities for safer practice and for more informative future research.

## Author Contributions

**Mohamed Khaldoun Badawy:** conceptualization, methodology, supervision, validation, writing – original draft, writing – review and editing. **Fatimah Balasim:** data curation, investigation, writing – review and editing. **Caitlan De Hoedt:** data curation, investigation, writing – review and editing. **Mercer Kim:** methodology, writing – review and editing. **Samuel Montgomery Lilli:** conceptualization, supervision, writing – review and editing. **Alice Gutowski:** conceptualization, supervision, writing – review and editing.

## Funding

This research received no specific grant from any funding agency in the public, commercial or not‐for‐profit sectors.

## Conflicts of Interest

M.K.B. is a member of the Editorial Board of the Journal of Medical Imaging and Radiation Oncology. In accordance with the journal's policy on submissions from members of the Editorial Board, he was excluded from the peer‐review process and from all editorial decision‐making regarding this manuscript. The authors declare no other competing interests and no financial conflicts of interest.

## Supporting information


**Appendix S1:** A priori systematic review protocol.

**Appendix S2:** Full database search strategies for Ovid MEDLINE(R) ALL, Embase, Scopus and the Cochrane Library.

**Table S1:** JBI critical‐appraisal (risk‐of‐bias) results for all included studies.

**Table S2:** Full data‐extraction dataset for all 25 included studies.

## Data Availability

The data supporting the findings of this review are available within the article and its Supporting Information. All data were extracted from previously published studies, which are publicly available and cited in the reference list; the complete data‐extraction dataset is provided as Table S2.

## References

[1] R. S. Nunna , F. Tariq , F. Jummah , N. Bains , A. I. Qureshi , and F. Siddiq , “Advances in the Endovascular Management of Cerebrovascular Disease,” Missouri Medicine 121, no. 2 (2024): 127–135. 38694595 PMC11057869

[2] S. Balter , J. W. Hopewell , D. L. Miller , L. K. Wagner , and M. J. Zelefsky , “Fluoroscopically Guided Interventions: A Review of Radiation Effects on Patients' Skin and Hair,” Radiology 254, no. 2 (2010): 326–341, 10.1148/radiol.2542082312. 20093507

[3] W. Jaschke , M. Schmuth , A. Trianni , and G. Bartal , “Radiation‐Induced Skin Injuries to Patients: What the Interventional Radiologist Needs to Know,” Cardiovascular and Interventional Radiology 40, no. 8 (2017): 1131–1140, 10.1007/s00270-017-1674-5. 28497187 PMC5489635

[4] D. Carrick , V. Carraro do Nascimento , L. de Villiers , and H. Rice , “Association of Radiation‐Induced Epilation and Interventional Neuroradiology Procedures,” Journal of Medical Imaging and Radiation Oncology 68, no. 7 (2024): 787–795, 10.1111/1754-9485.13730. 39054930

[5] ICRP , “ICRP Publication 118: ICRP Statement on Tissue Reactions and Early and Late Effects of Radiation in Normal Tissues and Organs – Threshold Doses for Tissue Reactions in a Radiation Protection Context,” Annals of the ICRP 41, no. 1–2 (2012): 1–322, 10.1016/j.icrp.2012.02.001. 22925378

[6] K. Riabroi , K. Khanungwanitkul , P. Wattanapongpitak , A. Krisanachinda , and K. Hongsakul , “Patient Radiation Dose in Neurointerventional Radiologic Procedure: A Tertiary Care Experience,” Neurointervention 13, no. 2 (2018): 110–116, 10.5469/neuroint.2018.01018. 30196681 PMC6132035

[7] M. J. Page , J. E. McKenzie , P. M. Bossuyt , et al., “The PRISMA 2020 Statement: An Updated Guideline for Reporting Systematic Reviews,” BMJ 372 (2021): n71, 10.1136/bmj.n71. 33782057 PMC8005924

[8] S. Moola , Z. Munn , C. Tufanaru , et al., “Chapter 7: Systematic Reviews of Etiology and Risk,” in JBI Manual for Evidence Synthesis, ed. E. Aromataris and Z. Munn (JBI, 2020), 10.46658/JBIMES-20-08.

[9] M. Campbell , J. E. McKenzie , A. Sowden , et al., “Synthesis Without Meta‐Analysis (SWiM) in Systematic Reviews: Reporting Guideline,” BMJ 368 (2020): l6890, 10.1136/bmj.l6890. 31948937 PMC7190266

[10] B. Allen , D. Cuzzone , C. Rowin , G. Perdrizet , and A. Babigian , “Fluoroscopic Radiation Burn After Embolization of a Spinal Arteriovenous Malformation,” Journal of Burn Care & Research 30, no. 2 (2009): 349–351, 10.1097/BCR.0b013e318198a710. 19165100

[11] G. J. Carstens , M. B. Horowitz , P. D. Purdy , and A. G. Pandya , “Radiation Dermatitis After Spinal Arteriovenous Malformation Embolization: Case Report,” Neuroradiology 38, no. Suppl 1 (1996): S160–S164, 10.1007/BF02278147. 8811705

[12] X. Chen , Y. Xie , and A. Wei , “Combined Corticosteroid and Minoxidil Therapy for Early Recovery in Radiation‐Induced Alopecia: A Case Report,” Clinical, Cosmetic and Investigational Dermatology 18 (2025): 1495–1499, 10.2147/CCID.S520575. 40546991 PMC12182084

[13] D. H. Choi , C. J. Yoo , C. W. Park , and M. J. Kim , “Practical Dose Parameter Values for the Prediction of the Adverse Effect of Neurointerventional Radiation: Relationship Between the Dose Parameters and Temporary Alopecia After Intracranial Coil Embolization,” World Neurosurgery 130 (2019): e222–e229, 10.1016/j.wneu.2019.06.039. 31203064

[14] S. Cho , M. J. Choi , J. S. Lee , Z. Zheng , and D. Y. Kim , “Dermoscopic Findings in Radiation‐Induced Alopecia After Angioembolization,” Dermatology 229, no. 2 (2014): 141–145, 10.1159/000362810. 25171463

[15] R. S. Corrigall , C. J. Martin , and I. Scott , “Observations of Tissue Reactions Following Neuroradiology Interventional Procedures,” Journal of Radiological Protection 40, no. 1 (2020): N9–N17, 10.1088/1361-6498/ab5bf4. 31770725

[16] M. Hayakawa , T. Moritake , F. Kataoka , et al., “Direct Measurement of Patient's Entrance Skin Dose During Neurointerventional Procedure to Avoid Further Radiation‐Induced Skin Injuries,” Clinical Neurology and Neurosurgery 112, no. 6 (2010): 530–536, 10.1016/j.clineuro.2010.03.019. 20392560

[17] W. Huda and K. R. Peters , “Radiation‐Induced Temporary Epilation After a Neuroradiologically Guided Embolization Procedure,” Radiology 193, no. 3 (1994): 642–644, 10.1148/radiology.193.3.7972801. 7972801

[18] T. Ishiguro , T. Satow , E. Hamano , et al., “Outcome of Endovascular Therapy Aiming for Single‐Session Obliteration of Intracranial Dural Arteriovenous Fistulas,” Neurologia Medico‐Chirurgica (Tokyo) 61, no. 10 (2021): 563–569, 10.2176/nmc.oa.2021-0059. PMC8531874 34148944

[19] R. S. Livingstone , L. Raghuram , I. P. Korah , and D. V. Raj , “Evaluation of Radiation Risk and Work Practices During Cerebral Interventions,” Journal of Radiological Protection 23, no. 3 (2003): 327–336, 10.1088/0952-4746/23/3/008. 14582723

[20] D. López Aventín , I. Gil , D. Manzano López González , and R. M. Pujol , “Chronic Scalp Ulceration as a Late Complication of Fluoroscopically Guided Cerebral Aneurysm Embolization,” Dermatology 224, no. 3 (2012): 198–203, 10.1159/000338891. 22677971

[21] V. López , I. López , and J. M. Ricart , “Temporary Alopecia After Embolization of an Arteriovenous Malformation,” Dermatology Online Journal 18, no. 9 (2012): 14, 10.5070/D31hq1006t. 23031381

[22] X. Lv , C. Jiang , Y. Li , M. Lv , and Z. Wu , “Endovascular Treatment of Dural Fistulas With the Venous Outflow of Laterocavernous Sinus,” European Journal of Radiology 75, no. 1 (2010): e129–e134, 10.1016/j.ejrad.2010.02.003. 20193996

[23] E. Marinello , F. Causin , M. B. Brumana , and M. Alaibac , “Radiodermatitis After Spinal Arteriovenous Fistula Embolisation,” BMJ~50% above 4.5 Gy Case Reports 2016 (2016): bcr2016214384, 10.1136/bcr-2016-214384. PMC4885348 27166008

[24] N. Martí , V. López , C. Pereda , J. M. Martín , E. Montesinos , and E. Jordá , “Radiation‐Induced Temporary Alopecia After Embolization of Cerebral Aneurysms,” Dermatology Online Journal 14, no. 7 (2008): 19, 10.5070/D389x969hk. 18718203

[25] R. B. Mooney , C. S. McKinstry , and H. A. M. Kamel , “Absorbed Dose and Deterministic Effects to Patients From Interventional Neuroradiology,” British Journal of Radiology 73, no. 871 (2000): 745–751, 10.1259/bjr.73.871.11089467. 11089467

[26] R. Nannapaneni , S. Behari , A. D. Mendelow , and A. Gholkar , “Temporary Alopecia After Subarachnoid Haemorrhage,” Journal of Clinical Neuroscience 14, no. 2 (2007): 157–161, 10.1016/j.jocn.2005.12.031. 17107802

[27] B. C. Perry , C. R. Ingraham , B. K. Stewart , K. Valji , and K. M. Kanal , “Monitoring and Follow‐Up of High Radiation Dose Cases in Interventional Radiology,” Academic Radiology 26, no. 2 (2019): 163–169, 10.1016/j.acra.2018.05.020. 29934019

[28] E. C. Peterson , K. M. Kanal , R. L. Dickinson , B. K. Stewart , and L. J. Kim , “Radiation‐Induced Complications in Endovascular Neurosurgery: Incidence of Skin Effects and the Feasibility of Estimating Risk of Future Tumor Formation,” Neurosurgery 72, no. 4 (2013): 566–572, 10.1227/NEU.0b013e318283c9a5. 23269458

[29] S. Suzuki , S. Furui , Y. Matsumaru , et al., “Patient Skin Dose During Neuroembolization by Multiple‐Point Measurement Using a Radiosensitive Indicator,” AJNR. American Journal of Neuroradiology 29, no. 6 (2008): 1076–1081, 10.3174/ajnr.A1045. 18388215 PMC8118850

[30] J. D. Thorat and P. Y. Hwang , “Peculiar Geometric Alopecia and Trigeminal Nerve Dysfunction in a Patient After Guglielmi Detachable Coil Embolization of a Ruptured Aneurysm,” Journal of Stroke and Cerebrovascular Diseases 16, no. 1 (2007): 40–42, 10.1016/j.jstrokecerebrovasdis.2006.10.003. 17689391

[31] M. Vaccaro , F. Guarneri , P. Brianti , and S. P. Cannavò , “Temporary Radiation‐Induced Alopecia After Embolization of a Cerebral Arteriovenous Malformation,” Clinical and Experimental Dermatology 40, no. 1 (2015): 88–90, 10.1111/ced.12472. 25284057

[32] C. S. Wen , S. M. Lin , Y. Chen , J. C. Chen , Y. H. Wang , and S. H. Tseng , “Radiation‐Induced Temporary Alopecia After Embolization of Cerebral Arteriovenous Malformations,” Clinical Neurology and Neurosurgery 105, no. 3 (2003): 215–217, 10.1016/S0303-8467(03)00007-6. 12860517

[33] P. Zhang and W. H. Fu , “Reversible Localized Alopecia After Angioplasty of Basilar Artery Stenosis in a Diabetic Patient: A Case Report,” SN Comprehensive Clinical Medicine 2, no. 8 (2020): 1198–1201, 10.1007/s42399-020-00386-x.

[34] National Council on Radiation Protection and Measurements , Radiation Dose Management for Fluoroscopically‐Guided Interventional Medical Procedures. NCRP Report No. 168 (NCRP, 2010).

[35] A. K. Jones and A. S. Pasciak , “Calculating the Peak Skin Dose Resulting From Fluoroscopically Guided Interventions. Part I: Methods,” Journal of Applied Clinical Medical Physics 12, no. 4 (2011): 3670, 10.1120/jacmp.v12i4.3670. 22089023 PMC5718743

[36] A. K. Jones and A. S. Pasciak , “Calculating the Peak Skin Dose Resulting From Fluoroscopically Guided Interventions. Part II: Case Studies,” Journal of Applied Clinical Medical Physics 13, no. 1 (2012): 174–186, 10.1120/jacmp.v13i1.3693. PMC5716137 22231220

