# Supplementary material for: Radiation‐Induced Cutaneous Tissue Reactions in Neurointerventional Procedures: A Systematic Review of Reported Effects and Associated Dose Levels
Source: J Med Imaging Radiat Oncol. 2026 Jul 30;70(6):561–72. doi: 10.1111/1754-9485.70156 (PMC13580428; doi:10.1111/1754-9485.70156)
Supplement: Supplementary file 1 [file ARA-70-561-s001.docx]

| **Database** | **Search** |
| --- | --- |
| MEDLINE OVID | Ovid MEDLINE(R) ALL <1946 to April 14, 2026>  1 exp alopecia/ 18514  2 (alopecia or epilation or radiodermatitis or erythema or hair loss or hair thinning or anagen effluvium or radiation burn* or (radiation adj3 skin injur*) or (radiation adj3 skin reaction*)).ti,ab,kw. 69724  3 1 or 2 74267  4 exp fluoroscopy/ or embolization/ 25507  5 (emboli?ation* adj5 (fistula* or arteriovenous malformation* or cerebral or intracranial or cerebrovascular or brain or aneurysm)).ti,ab,kw. 9555  6 (intracranial aneurysm* or arteriovenous malformation* or myelography or fluoroscop* or endovascular or emboliz* or embolisation or digital subtraction angiograph* or DSA or interventional neuroradiology or neuroradiolog* or neuro-radiolog* or cerebral angiograph*).ti,ab,kw. 239875  7 4 or 5 or 6 251209  8 3 and 7 367  9 limit 8 to (english language and humans) 255 |
| EMBASE | Embase <1974 to 2026 April 13>  1 exp alopecia/ 68327  2 (alopecia or epilation or radiodermatitis or erythema or hair loss or hair thinning or anagen effluvium or radiation burn* or (radiation adj3 skin injur*) or (radiation adj3 skin reaction*)).ti,ab,kw. 107935  3 1 or 2 144576  4 exp fluoroscopy/ or embolization/ 161314  5 (emboli?ation* adj5 (fistula* or arteriovenous malformation* or cerebral or intracranial or cerebrovascular or brain or aneurysm)).ti,ab,kw. 13943  6 (intracranial aneurysm* or arteriovenous malformation* or myelography or fluoroscop* or endovascular or emboliz* or embolisation or digital subtraction angiograph* or DSA or interventional neuroradiology or neuroradiolog* or neuro-radiolog* or cerebral angiograph*).ti,ab,kw. 366142  7 4 or 5 or 6 416766  8 3 and 7 891  9 limit 8 to (human and english language) 768 |
| SCOPUS | Scopus (searched April 2026)  ( TITLE-ABS-KEY ( alopecia OR epilation OR radiodermatitis OR erythema OR "hair loss" OR "hair thinning" OR "anagen effluvium" OR "radiation burn*" ) OR TITLE-ABS-KEY ( "radiation" W/3 "skin injur*" ) OR TITLE-ABS-KEY ( "radiation" W/3 "skin reaction*" ) ) AND ( TITLE-ABS-KEY ( "emboli?ation*" W/5 ( fistula* OR "arteriovenous malformation*" OR cerebral OR intracranial OR cerebrovascular OR brain OR aneurysm ) ) OR TITLE-ABS-KEY ( "intracranial aneurysm*" OR "arteriovenous malformation*" OR myelography OR fluoroscop* OR endovascular OR emboliz* OR embolisation OR "digital subtraction angiograph*" OR DSA OR "interventional neuroradiology" OR neuroradiolog* OR "neuro-radiolog*" OR "cerebral angiograph*" ) ) AND ( LIMIT-TO ( LANGUAGE , "English" ) ) AND ( LIMIT-TO ( EXACTKEYWORD , "Human" ) )  1,249 |
| Cochrane | Date run: 15 April 2026  ID Search Hits  #1 MeSH descriptor: [Alopecia] explode all trees 1218  #2 ((alopecia OR epilation OR radiodermatitis OR erythema OR hair loss OR hair thinning OR anagen effluvium OR radiation burn* OR (radiation NEAR/3 skin injur*) OR (radiation NEAR/3 skin reaction*))):ti,ab,kw (Word variations have been searched) 18604  #3 #1 or #2 18604  #4 MeSH descriptor: [Fluoroscopy] explode all trees 976  #5 MeSH descriptor: [Embolization, Therapeutic] explode all trees 1350  #6 ((emboli?ation* NEAR/5 (fistula* OR arteriovenous malformation* OR cerebral OR intracranial OR cerebrovascular OR brain OR aneurysm))):ti,ab,kw (Word variations have been searched) 485  #7 ((intracranial aneurysm* or arteriovenous malformation* or myelography or fluoroscop* or endovascular or emboliz* or embolisation or digital subtraction angiograph* or DSA or interventional neuroradiology or neuroradiolog* or neuro-radiolog* or cerebral angiograph*)):ti,ab,kw (Word variations have been searched) 28309  #8 #4 or #5 or #6 or #7 28818  #9 #3 AND #8 235 |
