# Supplementary material for: Radiation‐Induced Cutaneous Tissue Reactions in Neurointerventional Procedures: A Systematic Review of Reported Effects and Associated Dose Levels
Source: J Med Imaging Radiat Oncol. 2026 Jul 30;70(6):561–72. doi: 10.1111/1754-9485.70156 (PMC13580428; doi:10.1111/1754-9485.70156)
Supplement: Supplementary file 2 [file ARA-70-561-s003.docx]

Systematic Review Protocol

*Supplementary Material: a priori protocol*

# Administrative details

**1. Review title**

Radiation-induced cutaneous tissue reactions in neurointerventional procedures: a systematic review of reported effects and associated dose levels

**2. Original language title**

Not applicable (English).

**3. Anticipated or actual start date**

Project commenced February 2026; database searches conducted April 2026; protocol finalised before title/abstract screening.

**4. Anticipated completion date**

2026.

**5. Stage of review at time of this submission**

Preliminary searches: Completed

Piloting of the study selection process: Completed

Formal screening of search results against eligibility criteria: Completed

Data extraction: Completed

Risk of bias (quality) assessment: Completed

Data analysis: Completed

**6. Named contact**

Mohamed Khaldoun Badawy.

**7. Named contact email**

mohamed.badawy@monashhealth.org

**8. Organisational affiliation of the review**

Monash Health, Victoria, Australia, in collaboration with RMIT University, Melbourne, Australia.

**9. Named contact address**

Monash Radiology, Monash Health, 246 Clayton Road, Clayton VIC 3168, Australia.

**10. Review team members and their organisational affiliations**

Mohamed Khaldoun Badawy, Monash Radiology, Monash Health; Department of Medical Imaging and Radiation Sciences, Monash University.

Fatimah Balasim, Monash Radiology, Monash Health; School of Health and Biomedical Sciences, RMIT University.

Caitlan De Hoedt, Monash Radiology, Monash Health; School of Health and Biomedical Sciences, RMIT University.

Samuel Montgomery Lilli, Monash Radiology, Monash Health.

Alice Gutowski, Monash Radiology, Monash Health.

Mercer Kim, Library Services, Monash Health.

**11. Funding sources / sponsors**

None.

**12. Conflicts of interest**

M.K. Badawy is a member of the JMIRO Editorial Board; the authors declare no other competing interests.

**13. Collaborators**

Search strategy developed by the review team's medical librarians (co-author M. Kim).

# Review methods

**14. Review question(s)**

1. What radiation-induced cutaneous tissue reactions have been reported in patients undergoing neurointerventional procedures?

2. What radiation dose levels are associated with these reactions?

3. What is the time to onset of these reactions?

4. Which neurointerventional procedures are most commonly associated with these reactions?

**15. Searches**

Databases: Ovid MEDLINE(R) ALL (1946–14 April 2026); Embase (1974–13 April 2026); Scopus; Cochrane Library. Searched April 2026.

Two concept blocks combined with AND: (i) cutaneous radiation effects (alopecia, epilation, radiodermatitis, erythema, hair loss/thinning, anagen effluvium, radiation burn, radiation skin injury/reaction); (ii) neurointervention (fluoroscopy, embolisation of fistula/AVM/aneurysm/cerebral/intracranial/cerebrovascular lesions, DSA, endovascular, interventional neuroradiology, cerebral angiography).

Limits: English language; human. Search developed with a medical librarian. Full strategies provided as Supplementary Material.

**16. URL to search strategy**

Provided as Supplementary Material (full database search strategies).

**17. Condition or domain being studied**

Radiation-induced cutaneous (skin and hair) tissue reactions (deterministic effects of ionising radiation) following fluoroscopy- or DSA-guided neurointerventional (endovascular) procedures.

**18. Participants / population**

Inclusion: adult patients undergoing fluoroscopy- or DSA-guided cranial or spinal neurointerventional (endovascular) procedures (e.g. aneurysm coiling, AVM and dural/spinal arteriovenous fistula embolisation, intracranial angioplasty/stenting).

Exclusion: paediatric patients; animal or phantom studies; non-neurointerventional procedures; procedures not fluoroscopy/DSA-guided; radiation-therapy studies; spinal-pain/orthopaedic procedures (vertebroplasty, kyphoplasty, facet/epidural injections, nerve-root blocks).

**19. Intervention(s) / exposure(s)**

Exposure to ionising radiation during fluoroscopy- or DSA-guided neurointerventional procedures, characterised where reported by peak/entrance skin dose, reference-point/cumulative air kerma, or dose–area product.

**20. Comparator(s) / control**

None required. Where reported, patients with versus without cutaneous reactions, or across radiation-dose strata, are compared descriptively.

**21. Types of study to be included**

Include: case reports, case series, and observational studies (prospective or retrospective cohorts). Peer-reviewed; English language.

Exclude: narrative/literature reviews, commentaries, editorials, conference abstracts; non-peer-reviewed sources; studies reporting only brain or lens dose, or dose with no cutaneous outcome.

**22. Context**

Cranial and spinal neurointerventional radiology; any country; any year (no date restriction).

**23. Main outcome(s)**

(i) Type of radiation-induced cutaneous tissue reaction (alopecia/epilation, erythema/radiodermatitis, desquamation, ulceration/necrosis).

(ii) Associated radiation dose (by reported metric).

Measurement/timing: as reported in each study; any follow-up interval.

**24. Additional outcome(s)**

Time to onset; reversibility/permanence; neurointerventional procedure type associated with reactions; dose metric and reporting method; patient/procedural modifying factors (e.g. diabetes, repeat/staged procedures, cumulative dose, equipment factors); management; follow-up adequacy.

**25. Data extraction (selection and coding)**

Records de-duplicated and screened in Covidence by two independent reviewers (title/abstract then full text); conflicts resolved by discussion or a third reviewer. Data were extracted on a piloted standardised form by two reviewers who reached agreement; the dataset was then checked against source publications by the supervising team as a third verification, with discrepancies corrected against the primary source.

**26. Risk of bias (quality) assessment**

Joanna Briggs Institute (JBI) critical appraisal checklists appropriate to design (case reports; case series; cohort/analytical observational studies). Two reviewers independently; disagreements resolved by consensus.

**27. Strategy for data synthesis**

Narrative synthesis (informed by SWiM guidance) with tabulation; no meta-analysis (clinical/methodological heterogeneity; predominance of case reports). Incidence/denominator-based estimates derived only from cohort studies and reported separately from case-level descriptive data. Doses reported by metric without conversion and compared descriptively with established deterministic thresholds (ICRP 118; Balter 2010).

**28. Analysis of subgroups or subsets**

By reaction type; by neurointerventional procedure type; by study design (cohort vs case report/series); by dose metric.

# General information

**29. Type and method of review**

Systematic review with narrative synthesis.

**30. Language**

English.

**31. Country**

Australia.

**32. Other registration details**

None.

**33. Reference / URL for published protocol**

None.

**34. Dissemination plans**

Peer-reviewed publication (target: Journal of Medical Imaging and Radiation Oncology).

**35. Keywords**

Radiation-induced alopecia; epilation; radiodermatitis; peak skin dose; entrance skin dose; air kerma; neurointerventional radiology; endovascular embolisation; fluoroscopy; deterministic effects.

**36. Details of any existing review of the same topic by the same authors**

None.

**37. Current review status**

Complete; manuscript prepared for submission.

**38. Additional information**

Marked heterogeneity in radiation dose metrics across the literature is anticipated and is itself an outcome of interest.

**39. Details of final report/publication**

Reported in the accompanying manuscript (submitted for publication).
